# Age at First Fracture and Later Fracture Risk in Older Adults Undergoing Osteoporosis Assessment

**DOI:** 10.1001/jamanetworkopen.2024.48208

**Published:** 2024-12-02

**Authors:** Carrie Ye, Suzanne N. Morin, Lisa M. Lix, Eugene V. McCloskey, Helena Johansson, Nicholas C. Harvey, John A. Kanis, William D. Leslie

**Affiliations:** 1 Department of Medicine, University of Alberta, Edmonton, Alberta, Canada; 2 Department of Medicine, McGill University, Montreal, Quebec, Canada; 3 Department of Medicine, University of Manitoba, Winnipeg, Manitoba, Canada; 4 Centre for Metabolic Bone Diseases, University of Sheffield Medical School, Sheffield, United Kingdom; 5 MRC Versus Arthritis Centre for Integrated Research in Musculoskeletal Ageing, Department of Oncology and Metabolism, University of Sheffield, Sheffield, United Kingdom; 6 MRC Lifecourse Epidemiology Centre, University of Southampton, Southampton, United Kingdom; 7 Mary McKillop Institute for Health Research, Australian Catholic University, Melbourne, Australia; 8 NIHR Southampton Biomedical Research Centre, University of Southampton and University Hospital Southampton NHS Foundation Trust, Southampton, United Kingdom

## Abstract

**Importance:**

Fragility fractures are often defined as those that occur after a certain age (eg, 40-50 years). Whether fractures occurring in early adulthood are equally associated with future fractures is unclear.

**Objective:**

To examine whether the age at which a prior fracture occurred is associated with future fracture risk.

**Design, Setting, and Participants:**

This observational, population-based cohort study included individuals from the Manitoba Bone Mineral Density Registry with a first bone mineral density (BMD) measurement between January 1, 1996, and March 31, 2018, with and without prior fracture in adulthood. Data analysis was completed between April 1, and May 31, 2023.

**Exposure:**

Individuals with fractures before their first dual-energy x-ray absorptiometry were stratified by the age at first fracture (10-year intervals from 20-29 to ≥80 years of age).

**Main Outcomes and Measures:**

Incident fractures occurring after dual-energy x-ray absorptiometry (index date) and before March 31, 2021, were identified using linked provincial administrative health data.

**Results:**

The cohort included 88 696 individuals (80 066 [90.3%] female; mean [SD] age, 64.6 [11.0] years) with a mean (SD) femoral neck T score of −1.4 (1.0). A total of 21 105 individuals (23.8%) had sustained a prior fracture at a mean (SD) age of 57.7 (13.6) years (range, 20.0-102.4 years) at the time of first prior fracture. During a mean (SD) of 9.0 (5.5) years of follow-up, incident fractures occurred in 13 239 individuals (14.6%), including 12 425 osteoporotic fractures (14.0%), 9440 major osteoporotic fractures (MOFs) (10.6%), and 3068 hip fractures (3.5%). The sex- and age-adjusted hazard ratios for all incident fractures, osteoporotic fractures, and MOFs, according to age at first fracture, were all significantly elevated, with point estimates ranging from 1.55 (95% CI, 1.28-1.88) to 4.07 (95% CI, 2.99-5.52). After adjusting for the additional covariates, the effect estimates were similar and remained significantly elevated, with point estimates ranging from fully adjusted hazard ratios of 1.51 (95% CI, 1.42-1.60) to 2.12 (95% CI, 1.67-2.71) across age categories. Sensitivity analyses examining age at last prior fracture and in those with multiple prior fractures showed similar results.

**Conclusions and Relevance:**

In this cohort study, fractures in adulthood were associated with future fractures regardless of the age at which they occurred. Thus, fractures in early adulthood should not be excluded when assessing an individual’s ongoing fracture risk.

## Key Points

**Question:**

Is the age at which a prior fracture occurs associated with future fracture risk?

**Findings:**

In this population-level cohort study of 88 696 individuals, including 21 105 individuals with prior fracture, fractures in adulthood were associated with future fractures regardless of the age at which they occurred. The sex- and age-adjusted hazard ratios for all incident fractures, osteoporotic fractures, and major osteoporotic fractures, according to age at first fracture, were all significantly elevated.

**Meaning:**

Fractures occurring at any age in adulthood, including in early adulthood, should not be excluded when assessing an individual’s ongoing fracture risk.

## Teaser

This cohort study of individuals with fractures in adulthood examines whether the age at which a prior fracture occurred is associated with future fracture risk.

## Introduction

Accurate identification of fracture risk is necessary to ensure that those at highest risk of future fractures are identified for treatments that can reduce the risk of fractures. All fracture risk tools, including the most widely used and validated fracture risk calculator, FRAX,^1^ consider prior fracture. Prior fracture is one of the strongest factors associated with future fracture, with a recent meta-analysis of primary data from 64 cohorts showing that a previous fracture history, compared with individuals without a prior fracture, was associated with nearly 2-fold increased risk of any clinical fracture.^2^

Many studies investigating prior fracture as a risk factor for subsequent fracture focus on those occurring after middle to late adulthood.^2,3,4^ Consequently, nearly all the major fracture risk calculators, except FRAX, have a lower age cutoff between 40 and 50 years when considering prior fractures.^5,6,7^ However, it remains unclear whether fractures occurring before 40 to 50 years of age should also be considered when assessing an individual’s future risk of fracture.

Few studies have examined the association of fractures occurring in early adulthood with future fracture risk.^8,9,10,11^ Of these few studies, most were conducted only in women, had small sample sizes, ascertained fractures by participant recall alone, and examined large age categories, such as premenopausal.^8,9,10^ One study examining both male and female participants showed equivocal results for the relative risk of subsequent fracture in a later age category if a fracture occurred in a previous age category, possibly limited by small sample size.^11^ Thus, it remains unclear whether fractures occurring at varying ages in adult life carry the same increased risk for subsequent fracture. The objective of this study was to examine whether the age at which a prior fracture occurred is associated with future fracture risk.

## Methods

### Study Population and Data Sources

We performed a retrospective cohort study of individuals in Manitoba, the fifth largest province in Canada by population, with an estimated population of 1.4 million residents in 2018.^12^ Each province in Canada, including Manitoba, has a single provincial public health care system that offers health services to nearly all residents. Residents are assigned a unique personal health identification number that links their information across various provincial administrative databases, providing data on health care use, diagnoses, and outcomes. The study was approved by the Health Research Ethics Board for the University of Manitoba and Manitoba’s Provincial Health Research Privacy Committee. Waiver of consent was granted by the Health Research Ethics Board for the University of Manitoba because all data were deidentified. This study followed the Strengthening the Reporting of Observational Studies in Epidemiology (STROBE) reporting guideline.

We included all individuals from the Manitoba Bone Mineral Density Registry with a first dual-energy x-ray absorptiometry (DXA) between January 1, 1996, and March 31, 2018. The date of first DXA was designated the index date. We limited the cohort to individuals aged 40 years or older on the index date and enrolled in the provincial health insurance program. The Manitoba Bone Mineral Density Program oversees all clinical bone mineral density (BMD) testing in the province, and the Manitoba Bone Mineral Density Registry maintains a database of all DXA results, along with clinical parameters for each registrant. This population-based database is nearly 100% complete and accurate.^13^ Criteria for testing include being female and older than 65 years. Men of any age and women younger than 65 years also qualify for testing in the presence of additional risk factors.^14^ Provincial administrative health data from March 1979 onward were linked for individuals identified in the Manitoba Bone Mineral Density Registry.

Patient demographics and coverage period were obtained from the provincial registration database (a list of individuals enrolled in the provincial health insurance program). The database did not collect data on race and ethnicity. Information on diagnosis codes associated with health care visits and procedures were obtained from physician claims using *International Classification of Diseases, Ninth Revision, Clinical Modification* (*ICD-9-CM*) codes, and hospital discharge abstracts using *ICD-9-CM* before 2004 and *International Classification of Diseases, Tenth Revision, Canadian Enhancements* (*ICD-10-CA*) from 2004 onward. Medication use was ascertained from the provincial pharmacy database, which records all medications dispensed in the outpatient setting.^15^ Deaths were ascertained from the Vital Statistics registry, which records all deaths that occurred in Manitoba.^16^

### Fracture Assessment

All clinical fractures occurring at or after the age of 20 years, except those involving the head, neck, hands, and feet, diagnosed between March 1, 1979, and March 31, 2018, were ascertained using *ICD-9-CM* and *ICD-10-CA* codes from hospital discharge abstracts and physician billing claims. Fracture site–specific algorithms used have been previously validated against radiographic imaging results in individuals with and without fracture in the Manitoba Bone Mineral Density Registry.^17,18^ Hip and forearm fractures required site-specific reduction, fixation, or casting codes.^18^ Incident fracture date was defined as the date of the first clinical encounter for the fracture with no coding of the same fracture type allowed within 6 months preceding an incident fracture. Fractures associated with high trauma codes were excluded.

First prior fracture was defined as the first clinical fracture diagnosed between March 1, 1979, and the index date and was categorized by the age at first fracture into 10-year intervals from 20 to 29 years of age to 80 years or older. Incident fractures were defined as fractures diagnosed between the index date and March 1, 2018, and were further categorized as osteoporotic fractures if the fracture occurred at a site other than the ankle and major osteoporotic fractures (MOFs) if the fracture occurred at the hip, vertebrae, forearm, or humerus.

### Covariates

Age, sex, body mass index, parental hip fracture, femoral neck T score, and smoking status were ascertained from the Manitoba Bone Mineral Density Registry on the index date. Body mass index was calculated as weight in kilograms divided by height in meters squared. Parental hip fracture, smoking, rheumatoid arthritis, and high alcohol use were ascertained from the intake questionnaire and supplemented through linked administrative health care *ICD-9-CM* and *ICD-10-CA* codes. Secondary osteoporosis included an extensive list of conditions that can cause osteoporosis.^19^

Use of medications, including glucocorticoids and antiosteoporosis medications, was ascertained from the provincial pharmacy system. Prolonged glucocorticoid use was defined as dispensation of oral glucocorticoids of greater than 90 days in the prior year. Antiosteoporosis treatment was defined as dispensation of at least 180 days of medication in the prior year of any of the following: etidronate, alendronate, risedronate, zoledronic acid, raloxifene, calcitonin, denosumab, or teriparatide.

### Statistical Analysis

Analysis was completed between April 1, and May 31, 2023. Baseline characteristics were summarized for the entire cohort using mean (SD) values for continuous variables or numbers (percentages) for dichotomous variables. First prior fracture, categorized by fracture site, was presented as percentages by age category of first prior fracture.

Multivariable Cox proportional hazards regression models estimated hazard ratios (HRs) and their 95% CIs for any incident fracture, osteoporotic fracture, MOFs, and hip fracture by age interval at first adult fracture before the index date compared with those without prior fracture. Proportional hazards were confirmed using Schoenfeld residuals. Models were adjusted for age and sex (age- and sex-adjusted models) and additionally adjusted for body mass index, parental hip fracture, smoking status, prolonged glucocorticoid use, rheumatoid arthritis, secondary osteoporosis, high alcohol use, antiosteoporosis treatment, and femoral neck T score ascertained at the index date (fully adjusted models). Linear trend across age categories was tested in the same adjusted models.

Effect modification by sex was assessed using the 2-way interaction sex × prior fracture. Sensitivity analysis was conducted examining the adjusted HRs (AHRs) in the same Cox proportional hazards regression models above but stratified by age of last prior fracture instead of age of first prior fracture. Subgroup analysis was conducted in those with single vs multiple prior fractures using age of first prior fracture or age of last prior fracture. *P* < .05 was considered statistically significant. Statistical analyses were conducted using SAS, version 9.4 (SAS Institute Inc).

## Results

**Figure 1.**
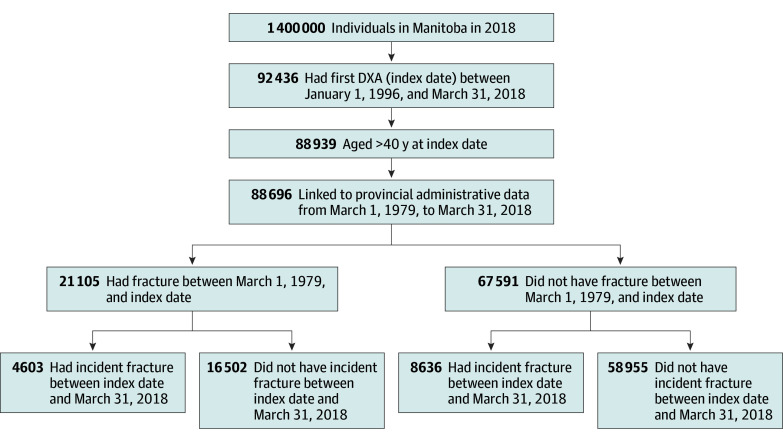
Flowchart of Fracture Incidence DXA indicates dual-energy x-ray absorptiometry.

A total of 92 436 individuals had a first DXA between January 1, 1996, and March 31, 2018, of whom 88 939 were 40 years or older on the index date (Figure 1). A total of 88 696 individuals (80 066 [90.3%] female and 8630 [9.7%] male; mean [SD] age, 64.6 [11.0] years) were enrolled in the provincial health insurance program and could be linked to provincial administrative data between March 1, 1979, and March 31, 2018 (Table 1). The mean (SD) femoral neck T score was −1.4 (1.0), 13 189 individuals (14.9%) had a secondary cause of osteoporosis, and 25 812 (29.1%) were receiving antiosteoporosis treatment. During a mean (SD) observation period of 25.1 (8.3) years before the index date, 21 105 (23.8%) had experienced at least 1 prior fracture, and 4639 (5.2% of the study cohort and 22.0% of those who had a prior fracture) had experienced more than 1 prior fracture. The mean (SD) age of first fracture before the index date was 57.7 (13.6) years (range, 20.0-102.4 years). During a mean (SD) follow-up period of 9.0 (5.5) years after the index date, 13 239 individuals (14.6%) had a clinical fracture, including 2245 (14.0%) who had osteoporotic fractures, 9440 (10.6%) who had MOFs, and 3068 (3.5%) who had hip fractures (Table 1).

**Table 1.** Baseline Characteristics and Incident Fractures Stratified by Prior Fracture

Characteristics	No. (%) of participants^a^		
	Overall (N = 88 696)	No prior fracture (n = 67 591)	Any prior fracture (n = 21 105)
Index age, mean (SD), y	64.6 (11.0)	63.9 (10.8)	66.9 (11.2)
Age group, y			
40-49	7608 (8.6)	6387 (9.4)	1221 (5.8)
50-59	22 722 (25.6)	18 042 (26.7)	4680 (22.2)
60-69	29 128 (32.8)	22 643 (33.5)	6485 (30.7)
70-79	20 421 (23.0)	14 798 (21.9)	5623 (26.6)
≥80	8817 (9.9)	5721 (8.5)	3096 (14.7)
Sex			
Female	80 063 (90.3)	61 493 (91.0)	18 570 (88.0)
Male	8630 (9.7)	6098 (10.0)	2535 (12.0)
BMI, mean (SD)	27.4 (8.0)	27.3 (8.4)	27.6 (6.6)
Parental hip fracture	6728 (7.6)	5095 (7.5)	1633 (7.7)
Smoker	8938 (10.1)	6304 (9.3)	2634 (12.5)
Prolonged glucocorticoid use	4841 (5.5)	3950 (5.8)	891 (4.2)
Rheumatoid arthritis	2781 (3.1)	2131 (3.2)	650 (3.1)
Secondary osteoporosis	13 189 (14.9)	10 212 (15.1)	2977 (14.1)
High alcohol use	528 (0.6)	261 (0.4)	267 (1.3)
Antiosteoporosis treatment	25 812 (29.1)	18 692 (27.7)	7120 (33.7)
Femoral neck T score, mean (SD)	−1.4 (1.0)	−1.3 (1.0)	−1.7 (1.0)
Femoral neck *z* score, mean (SD)	0.0 (1.0)	0.1 (1.0)	−0.2 (0.9)
Incident fracture	13 239 (14.9)	8636 (12.8)	4603 (21.8)
Osteoporotic fracture	12 425 (14.0)	8068 (11.9)	4357 (20.6)
Major osteoporotic fracture	9440 (10.6)	6108 (9.0)	3332 (15.8)
Hip fracture	3068 (3.5)	1896 (2.8)	1172 (5.6)
Observation time, mean (SD), y			
Before index DXA	25.1 (8.3)	24.6 (8.5)	26.4 (7.4)
After index DXA	9.0 (5.5)	9.3 (5.5)	8.3 (5.3)

Abbreviations: BMI, body mass index (calculated as weight in kilograms divided by height in meters squared); DXA, dual-energy x-ray absorptiometry.

^a^ Unless otherwise indicated.

**Figure 2.**
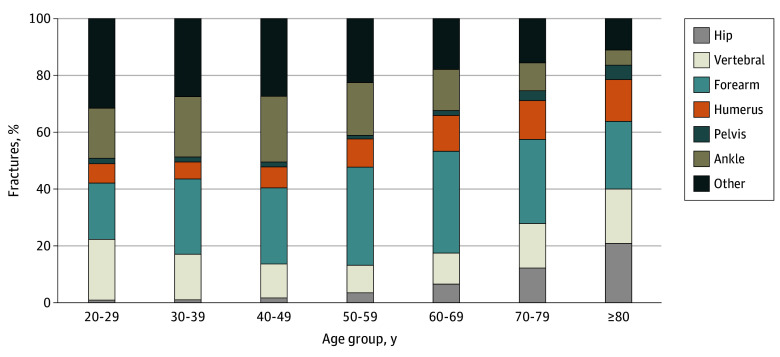
Age and Type of First Fracture Before First Dual-Energy X-Ray Absorptiometry Percentages of fracture type before the index date by age category at time of first fracture are shown.

The breakdown of first fracture site before the index date, by age category, is reported in eTable 1 in Supplement 1 and shown as percentages in Figure 2. The age category of 50 to 59 years had the greatest number of prior fractures (7617 [28.5%]), and the youngest age category, 20 to 29 years, had the least number of prior fractures (598 [2.2%]). Among those with prior fracture, 6654 (24.9%) occurred before 50 years of age and 2553 (9.5%) occurred before 40 years of age. Of the total 26 748 prior first fractures, the most common site of specific fracture before the index date in the full cohort was forearm fracture (8424 [31.5%]), followed by ankle fracture (4301 [16.1%]) and vertebral fracture (3303 [12.3%]). In the youngest age category (20-29 years), vertebral fractures were the most frequent specific type of fractures (128 of 598 [21.4%]), whereas in the oldest age category (≥80 years), forearm fractures were the most frequent fractures (385 of 1586 [24.3%]). Other fractures (those not falling into one of the specific categories of fractures) made up the largest percentage of fractures in the 3 youngest age categories (eTable 1 in Supplement 1). Most of the hip fractures occurred in the age group of 70 to 79 years, followed by the age group of 60 to 69 years, with these 2 age groups accounting for 962 of the 1683 total hip fractures (57.2%).

The sex- and age-adjusted HRs for all incident fractures, osteoporotic fractures, and MOFs, according to age at first fracture, were all significantly elevated (eTable 2 in Supplement 1), with point estimates ranging from 1.55 (95% CI, 1.28-1.88) to 4.07 (95% CI, 2.99-5.52). After adjusting for the additional covariates, the effect estimates were similar and remained significantly elevated, with point estimates ranging from fully AHRs of 1.51 (95% CI, 1.42-1.60) to 2.12 (95% CI, 1.67-2.71) across age categories (Table 2). The fully AHRs in these 3 categories of incident fractures were highest in the youngest age category (20-29 years), although there was no significant linear trend across age categories (Table 2). The fully AHRs for incident hip fracture were significantly elevated for all age categories except the youngest age category (Table 2). The largest fully AHR for incident hip fractures was observed in those with their first fracture occurring in the age category of 30 to 39 years, but again there was no statistically significant linear trend across age categories.

**Table 2.** Fully AHRs (95% CIs) for Incident Fracture According to Age at First Fracture Before the Index Date Compared With Those Without Prior Fracture^a^

First prior fracture age group, y	AHR (95% CI)			
	All fractures	Osteoporosis fractures	MOFs	Hip fractures
20-29	2.12 (1.67-2.71)	2.11 (1.63-2.74)	2.18 (1.61-2.95)	2.34 (0.97-5.65)
30-39	2.10 (1.86-2.37)	2.11 (1.86-2.40)	2.08 (1.79-2.42)	3.43 (2.52-4.67)
40-49	1.71 (1.57-1.86)	1.71 (1.56-1.87)	1.67 (1.51-1.85)	2.02 (1.64-2.48)
50-59	1.59 (1.50-1.69)	1.57 (1.48-1.67)	1.53 (1.43-1.64)	1.47 (1.29-1.67)
60-69	1.51 (1.42-1.60)	1.49 (1.40-1.59)	1.46 (1.36-1.56)	1.33 (1.18-1.49)
70-79	1.70 (1.58-1.83)	1.69 (1.57-1.83)	1.58 (1.45-1.72)	1.26 (1.11-1.44)
≥80	1.70 (1.50-1.92)	1.68 (1.49-1.90)	1.47 (1.28-1.70)	1.25 (1.03-1.51)
*P* value for trend^b^	.12	.30	.71	.16

Abbreviations: AHR, adjusted hazard ratio; MOFs, major osteoporotic fractures.

^a^ Adjusted for age at index date, sex, body mass index, parental hip fracture, smoker, prolonged glucocorticoid use, rheumatoid arthritis, secondary osteoporosis, high alcohol use, antiosteoporosis treatment, and femoral neck T score.

^b^ Linear trend across age categories.

Sex-stratified results are given in Table 3. The 95% CIs for male and female individuals overlapped but need to be interpreted with caution because 95% CIs were much wider in male than female individuals. The 2-way interaction of sex × prior fracture was not statistically significant in all analyses.

**Table 3.** Fully AHRs (95% CIs) for Incident Fracture According to Age at First Fracture Before the Index Date, Stratified by Sex, Compared With Those Without Prior Fracture^a^

First prior fracture age group, y	AHR (95% CI)							
	Female				Male			
	All fractures	Osteoporosis fractures	MOFs	Hip fractures	All fractures	Osteoporosis fractures	MOFs	Hip fractures
20-29	2.27 (1.73-2.97)	2.31 (1.73-3.07)	2.42 (1.74-3.37)	2.52 (0.94-6.75)	1.57 (0.89-2.75)	1.39 (0.76-2.57)	1.37 (0.64-2.95)	1.52 (0.21-11.20)
30-39	1.96 (1.71-2.24)	1.97 (1.71-2.27)	1.94 (1.64-2.29)	3.25 (2.31-4.58)	2.94 (2.18-3.97)	2.89 (2.12-3.95)	3.00 (2.08-4.34)	4.08 (1.97-8.44)
40-49	1.74 (1.59-1.90)	1.74 (1.58-1.91)	1.72 (1.54-1.91)	2.19 (1.77-2.72)	1.41 (1.07-1.86)	1.43 (1.08-1.89)	1.30 (0.92-1.84)	1.14 (0.54-2.38)
50-59	1.58 (1.49-1.68)	1.56 (1.47-1.67)	1.52 (1.42-1.64)	1.46 (1.27-1.67)	1.54 (1.22-1.95)	1.58 (1.25-2.00)	1.61 (1.23-2.12)	1.58 (0.96-2.62)
60-69	1.48 (1.39-1.58)	1.47 (1.38-1.56)	1.43 (1.33-1.54)	1.32 (1.17-1.48)	1.73 (1.39-2.15)	1.75 (1.40-2.18)	1.74 (1.35-2.24)	1.35 (0.85-2.14)
70-79	1.68 (1.55-1.82)	1.67 (1.54-1.81)	1.54 (1.41-1.68)	1.21 (1.05-1.39)	1.92 (1.51-2.45)	1.91 (1.50-2.44)	1.95 (1.48-2.55)	1.85 (1.23-2.78)
≥80	1.69 (1.48-1.92)	1.66 (1.45-1.89)	1.44 (1.24-1.68)	1.22 (0.99-1.49)	1.83 (1.28-2.62)	1.90 (1.33-2.72)	1.72 (1.15-2.59)	1.50 (0.81-2.76)
*P* value for sex × prior fracture interaction	NA	NA	NA	NA	.41	.41	.26	.95

Abbreviations: AHR, adjusted hazard ratio; MOFs, major osteoporotic fractures; NA, not applicable.

^a^ Adjusted for age at index date, sex, body mass index, parental hip fracture, smoker, prolonged glucocorticoid use, rheumatoid arthritis, secondary osteoporosis, high alcohol use, antiosteoporosis treatment, and femoral neck T score.

Sensitivity analysis was conducted assessing AHRs for incident fracture by age at last fracture instead of first fracture before the index date, and these analyses showed similar results (eTable 3 in Supplement 1). Subgroup analysis in those with single vs multiple fractures before the index date showed similar AHRs for incident fracture (eTable 4 in Supplement 1).

## Discussion

This observational cohort study showed that previous fractures occurring at any age during adulthood were associated with future fractures in older age. This finding contrasts with the commonly held notion that only adult fractures occurring at older ages are associated with increased risk of future fractures.^5,6,7,20^ Importantly, although the most commonly considered osteoporotic fractures (hip, vertebral, forearm, humerus, and pelvis) were the most frequent prior fractures to occur after 50 years of age, other fractures made up the largest percentage of fractures in the 3 youngest age categories. This finding underscores the importance of assessing fractures beyond MOFs, consistent with a meta-analysis of previous studies that found that any prior fracture was associated with a similar increase in fracture risk as prior MOFs.^21^ Although there was no statistically significant trend in AHRs for incident fracture with increasing age of first prior fracture, AHRs for incident fracture were slightly higher in the younger age categories, underscoring the importance of including fractures occurring in younger adults to avoid underestimating incident fracture risk.

A recent large meta-analysis (64 international cohorts) of the association of previous fracture with subsequent fracture confirmed that a previous history of fracture conferred a substantially increased risk of subsequent fracture, with findings similar in men and women.^2^ This study also examined the interaction of age at the time of assessment and prior fracture and found a decrease in the association of prior fracture with increasing age at the time of assessment. However, the investigators did not examine age at the time of prior fracture.

Few studies have examined the influence of age at the time of prior fracture. In one cross-sectional study of 1284 women with a mean age of 73 years, participants were asked to report their prior fracture history.^10^ This study found that fractures sustained between the ages of 20 and 50 years were associated with a 74% increase in the risk of fractures after the age of 50 years. A similar cross-sectional survey study of 12 162 women with a mean age of 52 years found that self-reported fractures sustained between 20 and 34 years of age increased the risk of fracture between 35 and 57 years of age, with an unadjusted HR of 1.9.^8^

One longitudinal study of 8632 women with a mean age of 72 years at enrollment examined self-reported premenopausal fractures after the age of 25 years and found that the adjusted HR of fracture in the 12-year observation period after enrollment was slightly elevated at 1.25 compared with those who did not have a premenopausal fracture.^9^ A similar longitudinal cohort study surveyed 934 male and 870 female participants at baseline for prior fractures and ascertained fractures occurring after enrollment through a computerized keyword search of all radiological reports. They categorized fractures into 5 age categories between 0 and 70 years and found that fractures occurring in one age group were associated with increased fracture risk in the subsequent age group although not necessarily in all age groups.^11^ These previous studies were likely prone to recall bias because prior fractures were usually assessed through patient questionnaires, limiting the ability to precisely estimate time since fracture, and most had a relatively small sample size. Moreover, subsequent fracture risk was assessed at arbitrarily assigned age intervals instead of at the time of clinical assessment when treatment decisions need to be made.

Most fracture risk prediction tools, aside from FRAX, only consider fractures occurring after a certain age.^5,6,7^ The Garvan Fracture Risk Calculator considers previous fractures occurring after the age of 50 years.^6^ The American Bone Health Fracture Risk Calculator only considers previous fractures occurring after 45 years of age,^7^ and the Canadian Association of Radiologists-Osteoporosis Canada tool only considers fractures that occurred after 40 years of age.^5^ In contrast, FRAX considers all previous low-trauma fractures occurring in adult life.^22^

### Strengths and Limitations

This study as several strengths, including the population-based design and large sample size. The cohort is representative of the types of patients who are referred for BMD assessment in clinical practice. Additionally, the criteria for BMD testing in Manitoba aligns with national BMD screening guidelines, strengthening the generalizability of these results.^23^ We had long observation periods before and after the index date and were able to ascertain incident fractures during these observation periods using fracture definitions that have been validated against radiographic review.^18^ This approach avoided recall bias, which can occur in survey studies, particularly with respect to fracture type and age that may have occurred decades earlier. Furthermore, using administrative health data in a setting where there is universal health coverage limits loss to follow-up.

This study also has some limitations. Although this cohort represents a clinical referral population, there is potential for selection bias toward those in whom health care professionals were concerned about fracture risk. Consequently, the limited number of fractures in male individuals may have limited our ability to reach statistical significance for the younger age groups of prior fracture, although all point estimates were greater than 1 and no significant effect modification by sex (interaction) was detected. Generalizability to the general population, particularly male individuals not referred for DXA, is unknown. Additionally, we did not explore childhood fractures (fractures occurring before the age of 20 years).

## Conclusions

In this cohort study, non–high-trauma fractures in adulthood across all decades were associated with future fractures regardless of the age at which they occurred. Thus, all non–high-trauma fractures occurring in adulthood, including during early adulthood, should be considered when assessing an individual’s future fracture risk.

## Notes

Supplement 1. **eTable 1.** Number of Individuals With a Fracture Prior to the Index Date, by Age Category at Time of First Fracture **eTable 2.** Age and Sex-Adjusted Hazard Ratios (HRs) With 95% Confidence Intervals (CIs) for Incident Fracture According to Age at First Fracture Prior to the Index Date, Compared to Those Without Prior Fracture **eTable 3.** Adjusted Hazard Ratios (aHRs) With 95% Confidence Intervals (CIs) for Incident Fracture According to Age at Last Fracture Prior to the Index Date, Compared to Those Without Prior Fracture **eTable 4.** Adjusted Hazard Ratios (aHRs) With 95% Confidence Intervals (CIs) for Incident Fracture According to Age at First Fracture Prior to the Index Date, in Those With Single Versus Multiple Fractures Prior to the Index Date, Compared to Those Without Prior Fracture

Supplement 2.
Data Sharing Statement
