# Supplementary material for: Age at First Fracture and Later Fracture Risk in Older Adults Undergoing Osteoporosis Assessment
Source: JAMA Netw Open. 2024 Dec 2;7(12):e2448208. doi: 10.1001/jamanetworkopen.2024.48208 (PMC11612869; doi:10.1001/jamanetworkopen.2024.48208)
Supplement: Supplement 1. — eTable 1. Number of Individuals With a Fracture Prior to the Index Date, by Age Category at Time of First Fracture eTable 2. Age and Sex-Adjusted Hazard Ratios (HRs) With 95% Confidence Intervals (CIs) for Incident Fracture According to Age at First Fracture Prior to the Index Date, Compared to Those Without Prior Fracture eTable 3. Adjusted Hazard Ratios (aHRs) With 95% Confidence Intervals (CIs) for Incident Fracture According to Age at Last Fracture Prior to the Index Date, Compared to Those Without Prior Fracture eTable 4. Adjusted Hazard Ratios (aHRs) With 95% Confidence Intervals (CIs) for Incident Fracture According to Age at First Fracture Prior to the Index Date, in Those With Single Versus Multiple Fractures Prior to the Index Date, Compared to Those Without Prior Fracture [file jamanetwopen-e2448208-s001.pdf]

Supplementary Online Content

Ye C, Morin SN, Lix LM, et al. Age at first fracture and later fracture risk in older adults undergoing osteoporosis assessment. *JAMA Netw Open*. 2024;7(12):e2448208.
doi:10.1001/jamanetworkopen.2024.48208

eTable 1. Number of Individuals with a Fracture Prior to the Index Date, by Age Category at Time of First Fracture
eTable 2. Age and Sex-Adjusted Hazard Ratios (HRs) With 95% Confidence Intervals (CIs) for Incident Fracture According to Age at First Fracture Prior to the Index Date, Compared to Those Without Prior Fracture
eTable 3. Adjusted Hazard Ratios[a] (aHRs) With 95% Confidence Intervals (CIs) for Incident Fracture According to Age at Last Fracture Prior to the Index Date, Compared to Those Without Prior Fracture
eTable 4. Adjusted Hazard Ratios[a] (aHRs) With 95% Confidence Intervals (CIs) for Incident Fracture According to Age at First Fracture Prior to the Index Date, in Those With Single Versus Multiple Fractures Prior to the Index Date, Compared to Those Without Prior Fracture

This supplementary material has been provided by the authors to give readers additional information about their work.

© 2024 Ye C et al. *JAMA Network Open.*

eTable 1. Number of Individuals with a Fracture Prior to the Index Date, by Age Category at Time of First Fracture

Age category	Hip	Vertebral	Forearm	Humerus	Pelvis	Ankle	Other	Total
20-29 years	6	128	118	40	12	104	190	598
30-39 years	25	311	518	117	31	417	536	1955
40-49 years	81	485	1092	304	70	941	1128	4101
50-59 years	276	734	2645	737	112	1399	1714	7617
60-69 years	469	733	2493	871	128	974	1254	6922
70-79 years	493	615	1173	548	130	388	622	3969
≥80 years	333	297	385	232	83	78	178	1586
All ages	1683	3303	8424	2849	566	4301	5622	26748

© 2024 Ye C et al. *JAMA Network Open.*

eTable 2. Age and Sex-Adjusted Hazard Ratios (HRs) with 95% Confidence Intervals (CIs) for Incident Fracture According to Age at First Fracture Prior to the Index Date, Compared to Those Without Prior Fracture

Age at last prior fracture	All fractures HR (95% CI)	OP fractures HR (95% CI)	MOF HR (95% CI)	Hip fractures HR (95% CI)
20-29 years	2.46 (1.93-3.13)	2.48 (1.92-3.21)	2.60 (1.92-3.52)	3.11 (1.29-7.53)
30-39 years	2.33 (2.07-2.64)	2.37 (2.08-2.69)	2.36 (2.03-2.75)	4.07 (2.99-5.52)
40-49 years	1.88 (1.73-2.05)	1.89 (1.74-2.07)	1.88 (1.70-2.08)	2.37 (1.93-2.91)
50-59 years	1.80 (1.70-1.91)	1.79 (1.69-1.91)	1.78 (1.66-1.91)	1.76 (1.55-2.00)
60-69-years	1.68 (1.58-1.79)	1.68 (1.58-1.79)	1.66 (1.55-1.78)	1.57 (1.40-1.75)
70-79 years	1.92 (1.78-2.07)	1.93 (1.79-2.08)	1.82 (1.67-1.98)	1.55 (1.36-1.77)
≥ 80 years	1.94 (1.71-2.19)	1.94 (1.72-2.20)	1.72 (1.50-1.99)	1.55 (1.28-1.88)

OP=osteoporosis; MOF=major osteoporotic fracture.

© 2024 Ye C et al. *JAMA Network Open.*

eTable 3. Adjusted Hazard Ratios[a] (aHRs) with 95% Confidence Intervals (CIs) for Incident Fracture According to Age at Last Fracture Prior to the Index Date, Compared to Those Without Prior Fracture

Age at last prior fracture	All fractures aHR (95% CI)	OP fractures aHR (95% CI)	MOF aHR (95% CI)	Hip fractures aHR (95% CI)
20-29 years	1.78 (1.31-2.42)	1.73 (1.24-2.40)	1.79 (1.21-2.63)	0.65 (0.09-4.61)
30-39 years	1.85 (1.60-2.13)	1.82 (1.57-2.12)	1.80 (1.51-2.15)	3.18 (2.21-4.56)
40-49 years	1.64 (1.49-1.79)	1.62 (1.47-1.78)	1.53 (1.37-1.71)	1.69 (1.33-2.15)
50-59 years	1.59 (1.49-1.69)	1.57 (1.48-1.68)	1.56 (1.45-1.67)	1.48 (1.29-1.71)
60-69-years	1.52 (1.44-1.62)	1.51 (1.42-1.61)	1.49 (1.39-1.59)	1.37 (1.22-1.53)
70-79 years	1.78 (1.66-1.91)	1.76 (1.64-1.89)	1.67 (1.54-1.80)	1.40 (1.24-1.58)
≥ 80 years	1.84 (1.65-2.05)	1.82 (1.64-2.03)	1.54 (1.36-1.75)	1.23 (1.03-1.46)

[a]Adjusted for age at index date, sex, body mass index, parental hip fracture, smoker, prolonged glucocorticoid use, rheumatoid arthritis, secondary osteoporosis, high alcohol use, anti-osteoporosis treatment, femoral neck T-score. OP=osteoporosis; MOF=major osteoporotic fracture.

© 2024 Ye C et al. *JAMA Network Open.*

eTable 4. Adjusted Hazard Ratios[a] (aHRs) with 95% Confidence Intervals (CIs) for Incident Fracture According to Age at First Fracture Prior to the Index Date, in Those with Single Versus Multiple Fractures Prior to the Index Date, Compared to Those Without Prior Fracture

Age at first prior fracture	All fractures aHR (95% CI)	OP fractures aHR (95% CI)	MOF aHR (95% CI)	Hip fractures aHR (95% CI)
Single prior fracture, N=16,466				
20-29 years	1.68 (1.21-2.34)	1.64 (1.15-2.34)	1.67 (1.1-2.54)	0.77 (0.11-5.47)
30-39 years	1.82 (1.56-2.13)	1.78 (1.51-2.10)	1.77 (1.46-2.15)	3.53 (2.43-5.13)
40-49 years	1.58 (1.43-1.75)	1.56 (1.41-1.74)	1.48 (1.30-1.67)	1.54 (1.17-2.02)
50-59 years	1.45 (1.35-1.55)	1.43 (1.33-1.54)	1.44 (1.32-1.56)	1.36 (1.16-1.59)
60-69-years	1.40 (1.31-1.50)	1.40 (1.30-1.50)	1.39 (1.29-1.51)	1.29 (1.14-1.47)
70-79 years	1.69 (1.56-1.83)	1.68 (1.55-1.82)	1.60 (1.46-1.76)	1.32 (1.15-1.52)
≥ 80 years	1.71 (1.50-1.95)	1.70 (1.49-1.94)	1.49 (1.28-1.74)	1.28 (1.04-1.58)
Multiple prior fracture, N=4,639				
20-29 years	3.25 (2.28-4.64)	3.28 (2.26-4.77)	3.46 (2.25-5.33)	5.38 (2.0-14.46)
30-39 years	2.89 (2.38-3.50)	3.00 (2.46-3.66)	2.91 (2.30-3.66)	3.54 (2.12-5.93)
40-49 years	2.14 (1.84-2.48)	2.17 (1.86-2.53)	2.27 (1.91-2.69)	3.46 (2.55-4.69)
50-59 years	2.06 (1.86-2.29)	2.02 (1.81-2.25)	1.83 (1.62-2.07)	1.73 (1.40-2.14)
60-69-years	1.91 (1.71-2.14)	1.84 (1.64-2.06)	1.66 (1.45-1.89)	1.39 (1.13-1.70)
70-79 years	1.75 (1.49-2.06)	1.76 (1.49-2.07)	1.43 (1.18-1.73)	1.01 (0.75-1.36)
≥ 80 years	1.65 (1.21-2.26)	1.60 (1.16-2.19)	1.39 (0.97-2.00)	1.01 (0.61-1.66)

[a]Adjusted for age at index date, sex, body mass index, parental hip fracture, smoker, prolonged glucocorticoid use, rheumatoid arthritis, secondary osteoporosis, high alcohol use, anti-osteoporosis treatment, femoral neck T-score. OP=osteoporosis; MOF=major osteoporotic fracture.

© 2024 Ye C et al. *JAMA Network Open.*
