# Supplementary material for: Age at First Fracture and Later Fracture Risk in Older Adults Undergoing Osteoporosis Assessment
Source: JAMA Netw Open. 2024 Dec 2;7(12):e2448208. doi: 10.1001/jamanetworkopen.2024.48208 (PMC11612869; doi:10.1001/jamanetworkopen.2024.48208)
Supplement: Supplement 2. — Data Sharing Statement [file jamanetwopen-e2448208-s002.pdf]

Data Sharing Statement

Ye. Age at First Fracture and Later Fracture Risk in Older Adults Undergoing Osteoporosis Assessment. *JAMA Netw Open*. Published December 02, 2024.
doi:10.1001/jamanetworkopen.2024.48208

Data
Data available: No
